# Striatal RGS7 Regulates Depression-Related Behaviors and Stress-Induced Reinstatement of Cocaine Conditioned Place Preference

**DOI:** 10.1523/ENEURO.0365-20.2020

**Published:** 2021-03-09

**Authors:** Laurie P. Sutton, Natalia Khalatyan, Jeffrey N. Savas, Kirill A. Martemyanov

**Affiliations:** 1 Department of Neuroscience, The Scripps Research Institute, Jupiter, FL 33458; 2 Department of Neurology, Northwestern University Feinberg School of Medicine, Chicago, IL 60611

**Keywords:** addiction, depression, reinstatement, Rgs, stress, striatum

## Abstract

The striatum plays a key role in both reward-related and affective behaviors and disruptions to this circuit contributes to depression and drug addiction. However, our understanding of the molecular factors that facilitate and modify these processes are incomplete. Striatal function is modulated by G-protein-coupled receptors (GPCRs) that process vast neuromodulatory inputs. GPCR signaling is negatively regulated by regulator of G-protein signaling (Rgs) proteins. In this study, we examine the role of striatal Rgs proteins in depressive-like and reward-related behaviors in male mice. Using a genetic mouse model with specific elimination of Rgs7 in striatal neurons we found that these mice exhibit an anxiolytic-like and antidepressant-like phenotype. In contrast, knock-out of Rgs9, an abundant Rgs protein in the same neuronal population did not affect the behavioral outcome in the depressive-like tests. Mice lacking striatal Rgs7 did not show significant differences in cocaine-induced psychomotor activation, sensitization or conditional place preference (CPP). Interestingly, loss of Rgs7 in the striatum made mice resilient to stress-induced but not drug-induced reinstatement of cocaine CPP. Analysis of striatal proteome revealed that loss of Rgs7 selectively affected expression of several networks, most prominently including proteins involved in translation and vesicular exocytosis. Together, these findings begin to demonstrate the specific contribution of Rgs7 acting in the striatum toward depression as it relates to stress-induced reinstatement of drug use.

## Significance Statement

G-protein-coupled receptors (GPCRs) play a key role in modulating responses of striatal neurons that ultimately shape complex behaviors such as mood and reward. The extent of GPCR signaling is tightly controlled by regulators of G-protein signaling (Rgs). In this study, we report a key role of Rgs7 in the striatum toward depression and reward-related behaviors, while addressing the effects of stress on these behavioral outcomes. Together, our findings provide new insights into the molecular mechanisms involved in stress induced drug seeking behaviors.

## Introduction

Converging human and rodent findings demonstrate a key role for the striatum in processing and responding to rewarding and aversive stimuli and is a critical mediator of affective states (Berton et al., 2006; Lobo and Nestler, 2011). The striatum serves as a central interface for integrating information from the ventral tegmental area (VTA) and prefrontal cortex (PFC) onto medium spiny neurons (MSNs). These afferent inputs onto MSNs lead to long-term adaptations in dendritic spine density, neuronal excitability and changes in gene expression which drive emotional and rewarding processes (Cerovic et al., 2013; Nelson and Kreitzer, 2014). Dysregulation of the striatal circuit contribute to several neuropsychiatric disorders including mood disorders and drug addiction (Lobo and Nestler, 2011; Francis and Lobo, 2017). Mood disorders have a high comorbidity with drug addiction which may stem from common molecular mechanisms (Pettinati et al., 2013). However, despite the relevance of the striatum in mediating reward and mood behaviors, we are just beginning to understand the neuroadaptations within the striatum that contribute to these neuropsychiatric disorders.

The activity of MSNs is controlled by multiple neurotransmitters, many of which act on their cognate G-protein-coupled receptor (GPCRs) to drive striatal-mediated behaviors (Kreitzer, 2009; Johnson and Lovinger, 2016). Activated GPCRs promote dissociation of the G-protein heterotrimer into Gβγ and the Gα-GTP subunits which trigger various cellular responses. To control the strength and duration of this signaling, regulator of G-protein signaling (RGS) proteins accelerate the inactivation of the Gα subunit promoting heterotrimer reformation (Ross and Wilkie, 2000; Hollinger and Hepler, 2002). In particular, a member of the RGS family, Rgs7 has been shown to play key roles in suppressing Gαi/o-mediated signaling via dopamine, opioid and adrenergic GPCRs thereby controlling mood and reward processes (Masuho et al., 2013; Sutton et al., 2016; Orlandi et al., 2019). Mice with a global knock-out of Rgs7 exhibit marked antidepressant-like behaviors and a resilience to chronic stress-induced depression (Orlandi et al., 2019). This phenotype can be suppressed by re-expression of Rgs7 within the PFC implicating this brain region in the effects. However, it remained unclear whether other neuronal populations and brain structures are involved in the effects of Rgs7 on affective behaviors in particular as it relates to addiction. In the striatum, Rgs7 has been implicated in dictating the sensitivity of mice to rewarding and reinforcing effects of morphine (Sutton et al., 2016). In this study we explore the role of striatal Rgs7 in depression related phenotypes and its relevance to regulating reward-related behaviors. We report that inactivation of Rgs7 specifically in striatal neurons results in prominent antidepressant-like effects and protects male mice from stress-induced but not drug-cued reinstatement of cocaine conditional place preference (CPP). Analysis of molecular changes suggest the involvement of complex gene networks in the observed phenotypes.

## Materials and Methods

### Animals

All studies were conducted in accordance with the National Institute of Health guidelines and were granted formal approval by the Institutional Animal Care and Use Committee. Conditional knock-out mice were generated by crossing homozygous *Rgs7^loxP/loxP^* with heterozygous *Rgs9^cre^* mice to generate *Rgs7^loxP/loxP^Rgs9^cre^* knock-out mice and their wild-type littermate control mice, *Rgs7^loxP/loxP^*(Dang et al., 2006; Cao et al., 2012). Generation of *Rgs9*−/− (Witherow et al., 2000) mice have been previously described. Mice were housed in groups on a 12/12 h light/dark cycle (lights on at 7:00 A.M.) with food and water available *ad libitum*. We relied exclusively on littermates for all the comparisons. Male mice were used in all the behavioral and biochemical assays and were between the ages of two to four months.

### Behavioral paradigms

#### Marble burying (MB)

MB was conducted in a homecage-like environment (27 × 16.5 × 12.5 cm) with 5-cm corncob bedding. Twenty glass marbles were overlaid in a 4 × 5 equidistant arrangement and testing consisted of a 30-min exploration period. The number of marbles that were at least two-thirds buried at the end of the trial were counted as buried.

#### Elevated plus maze (EPM)

The elevated plus maze was performed using a black, Plexiglas elevated plus maze (Med Associates). Lighting for the maze was set at 200 lux in the center of the plus maze, 270 lux on the open arms, and 120 lux on the closed arms. Testing consisted of 5-min exploration time and was recorded using Ethovision XT. The time spent in the open and closed arms and the number of entries from the closed to the open arm was calculated.

#### Forced swim test (FST)

The Porsolt FST was conducted using a vertical clear glass cylinder (10 cm in diameter, 25 cm in height) filled with water (25°C). The mice spent 6 min in the water, and immobility was scored from 2 to 6 min. Immobility was counted when the mouse floated motionless or made only those movements necessary to keep its head above the water.

#### Tail suspension test (TST)

The tails of the mice were wrapped with tape that covered ∼4/5 of the tail length and then fixed upside down on a hook. The immobility time of each mouse was recorded and tracked over a 6-min period using Ethovision XT.

#### Locomotion

Locomotor activity was performed in 40 × 40 × 35 chambers (Stoelting Co) and distance traveled was recorded using Anymaze video-tracking software. All mice were handled and inject with saline (intraperitoneal) for 3 d to minimize stress. Mice were randomly selected to be injected with saline or cocaine (15 mg/kg, i.p.) and placed in the center of the chambers. Distance traveled was measure for 3 h.

#### CPP, extinction, and reinstatement

CPP was conducted using a two-chamber box with a tunnel adjoining the chambers with each chamber distinguished by different color and floor textures (Stoelting Co). The CPP procedure consisted of four phases: habituation, preconditioning test, conditioning, and postconditioning test. On day 1, animals were habituated to the apparatus by allowing free access to all compartments for 10 min. The following day, all mice were exposed to 30 min preconditioning phase, where each animal was given free access to the CPP apparatus to assess whether animals had a bias to a given side. Mice that spent <70% of the time in any of the two chambers or tunnel were excluded from further evaluation. Subsequently, conditioning group (saline vs cocaine) and drug-chamber pairings, were pseudo-randomly assigned to achieve a balanced CPP design. During the 6 d of conditioning (days 3–8), animals were injected once a day with either vehicle or cocaine (4 or 10 mg/kg, i.p.) and immediately confined to one of the assigned compartments for 30 min. The order of the drug administration was counterbalanced such that half the animals received cocaine on the first day of conditioning and the other half on the second day of conditioning. On day 9, mice were placed in the center of the tunnel and allowed free access to all compartments for 30 min (postconditioning). Place preference score was calculated for each mouse as the difference between postconditioning and preconditioning time spent in drug-paired compartment. After conditioning, daily extinction training was conducted.

During the extinction sessions, mice were placed into the center compartment and once again provided free access to side compartments for 30 min. Mice underwent daily extinction training twice a day (morning and afternoon) until the preference for the cocaine paired compartment were similar to the preconditioning scores. Extinction was achieved when during the postextinguished test, the average preference for the cocaine paired compartment minus the standard error of the mean was below zero. Those mice that met the extinguished criteria underwent a reinstatement session. Reinstatement was performed the day following extinction. For stress-induced reinstatement mice were exposed to 6-min FST followed by 20-min recovery in a paper towel-lined cage and then a 30-min test in the CPP apparatus as above. For cocaine reinstatement, mice were injected with cocaine (10 mg/kg). Mice were then placed into the apparatus and allowed free access for 30 min. Reinstatement was defined according to the time spent in the compartment previously paired with cocaine. Time spent in each chamber was measured during each phase of the CPP using video tracking followed by the analysis by Anymaze Software.

### Quantitative proteomics and analysis

Ventral striatum (V. Str) and dorsal striatum (D. Str) for Rgs7 striatal knockout (sKO) and wildtype (WT) mice were homogenized and lysed in 6 m guanidine, 100 mm HEPES, pH 8.5, and prepared as previously described (He et al., 2019). Each sample was heated to 95°C for 3 min. The proteins were reduced at 5 mm DTT for 20 min and alkylated at 15 mm iodoacetamide for 20 min. The reaction was quenched by adding DTT to 50 mm and incubation for 15 min. Next, the solution was then diluted to 50 mm HEPES, 1.5 m guanidine; 1 μg of Lys-C protease (Pierce) was added to each sample and incubated for 3 h at room temperature while vortexing; 2 μg of trypsin protease (Pierce) was added next and samples were incubated overnight at 37°C while vortexing. Following digestion, the samples were acidified 0.5% TFA, bound to alkylated resin (Pierce C18 spin columns), and washed with 5% acetonitrile, 0.5% TFA. Samples were eluted from resin with 80% acetonitrile, 0.5% formic acid buffer. Eluted samples were dried down using vacuum centrifugation, and resuspended in 50 mm HEPES. MicroBCA (Pierce) was used to determine peptide mass concentration. A total of 80 μg of each sample was aliquoted for TMT labeling with 0.4 mg of a respective TMT label (Thermo Scientific). V. Str and D. Str samples were labeled as 5xCre– (WT) and 5xCre+ (Rgs7 sKO). Labeling reaction took place for 1 h and 15 min at room temperature. Reaction was quenched by bringing sample solutions to 0.3% (v/v) hydroxylamine and incubated for 15 min at room temperature. The ten samples for each brain region was then combined at a ratio of 1:1:1:1:1:1:1:1:1:1. The combined samples were then acidified to 0.5% TFA, bound to alkylated resin (HyperSep C18 vacuum cartridges), and washed with 5% acetonitrile, 0.5% TFA before being eluted with 80% acetonitrile, 0.5% formic acid. Eluted combinatory samples were dried down using vacuum centrifugation, and subsequently resuspended in 0.1% TFA. Samples were fractionated using strong cation exchange nitrocellulose spin columns (Pierce). Six elution fractions for each sample were created corresponding to 50 mm sodium acetate (NaAcO), 100 mm NaAcO, 250 mm NaAcO, 500 mm NaAcO, 1 m NaAcO, and 4 m NaAcO. Every fraction was desalted by acidification to pH 2 with TFA, binding to alkylated resin (Pierce C18 spin columns), washing with 5% acetonitrile, 0.5% TFA and eluted with 80% acetonitrile, 0.5% formic acid. Fractions were dried using vacuum centrifugation, and resuspended in liquid chromatography/mass spectrometry buffer A: 5% acetonitrile, 0.125% formic acid. Fractions were quantified using microBCA (Pierce); 3 μg from each fraction were loaded for LC-MS analysis using a Thermo Orbitrap Fusion coupled to a Thermo EASY nLC-1200 UPLC pump and vented Acclaim Pepmap 100, 75 μm × 2 cm nanoViper trap column and nanoViper analytical column: Thermo-164570, 3 μm, 100 Å, C18, 0.075 mm, 500 mm with stainless steel emitter tip assembled on the Nanospray Flex Ion Source with a spray voltage of 2000V. For the chromatographic run, buffer A contained (as above) and buffer B contained 95% acetonitrile, 0.125% formic acid. A 4-h gradient was established beginning with 100% A, 0% B, and increased to 7% B over 5 min, then to 25% B over 160 min, 36% B over 40 min, 45% B over 10 min, 95% B over 10 min, and held at 95% B for 15 min before terminating the scan. The multinotch MS3 method (McAlister et al., 2014) parameters include: ion transfer tube temperature = 300°C, Easy-IC internal mass calibration, default charge state = 2, and cycle time = 3 s. MS1 detector set to orbitrap with 60 K resolution, wide quad isolation, mass range = normal, scan range = 300–1800 m/z, max injection time = 50 ms, AGC target = 2 × 105, microscans = 1, RF lens = 60%, without source fragmentation, and datatype = positive and centroid. MIPS was set as on, included charge states 2–7 and reject unassigned. Dynamic exclusion was enabled with *n* = 1 exclusion for 60 s with 10 ppm tolerance for high and low. An intensity threshold was set to 5 × 103. Precursor selection decision = most intense, top speed, 3 s. MS2 settings include isolation window = 0.7, scan range = auto normal, collision energy = 35% CID, scan rate = turbo, max injection time = 50 ms, AGC target = 1 × 104, Q = 0.25. The top 10 precursors were selected for MS3 analysis. Precursors were fragmented using 65% HCD before orbitrap detection. A precursor selection range of 400–1200 m/z was chosen with mass range tolerance. An exclusion mass width was set to 18 ppm on the low and 5 ppm on the high. Isobaric tag loss exclusion was set to TMT reagent. Additional MS3 settings include an isolation window = 2, orbitrap resolution = 60 K, scan range = 120–500 m/z, AGC target = 1*104, max injection time = 120 ms, microscans = 1, and datatype = profile. Spectral raw files were extracted into MS1, MS2, and MS3 files using the in-house program RawConverter (He et al., 2015). Spectral files were pooled from fractions and an unfractionated portion for each sample and searched against the Uniprot mouse protein database (reviewed_iso_- cont_3_25_14) and matched to sequences using the Pro-LuCID/SEQUEST algorithm (ProLuCID version 3.1) with 50 ppm peptide mass tolerance for precursor ions and 600 ppm for fragment ions. The search space included all fully and half-tryptic peptide candidates that fell within the mass tolerance window with no miscleavage constraint, assembled, and filtered with DTASelect2 (version 2.1.3) through the Integrated Proteomics Pipeline (IP2 v.5.0.1, Integrated Proteomics Applications). Static modifications included 57.02,146 C and 229.162932 K and N-term. Peptide probabilities and false discovery ratios were produced using a target/decoy approach. Each protein identified was required to have a minimum of one peptide of minimal length five. A false discovery rate of 1% at the protein level was used for data filtering. Isobaric labeling analysis was performed with Census 2 as previously described (Park et al., 2014a). TMT channels were normalized by dividing it over the sum of all channels. No intensity threshold was applied.

To calculate the fold change between Rgs7 sKO and WT, the average intensity values for each protein in the dataset were used and the values were standardized to the mean of the WT samples (*n* = 5). The fold change was used to calculate the mean of the Rgs7 sKO standardized values, and the *p* values were calculated by a Student’s *t* test. For Panther analysis, the list of significantly changed proteins were queried against all proteins in the both the ventral and dorsal striatum dataset using a statistical overrepresentation test of the Gene Ontology (GO) biological process complete annotation (Mi et al., 2016).

### Western blottings

Brains were quickly removed from euthanized Rgs7 sKO and WT mice and striatal tissue was lysed in ice-cold lysis buffer [300 mm NaCl, 50 mm Tris-HCl, pH 7.4, 1% Triton X-100, and complete protease inhibitor cocktail (Roche Applied Science) and phosphatase inhibitor mix (Sigma-Aldrich)] and sonicated. Protein concentrations was obtained using Pierce 660 nm Protein Assay (Thermo Fisher Scientific). Samples were diluted in 4× SDS sample buffer, resolved by SDS-PAGE, and then transferred onto a polyvinylidene difluoride membrane. Primary antibodies for anti-RGS7 and anti-GAPDH (Millipore) were detected by using horseradish peroxidase-conjugated secondary antibodies and ECL chemiluminescence system (Pierce). Signals were captured on film and scanned by densitometer, and band intensities were determined by using NIH ImageJ software.

### Quantification and statistical analysis

Statistical analysis was performed using GraphPad Prism (Prism6.0, GraphPad). Student’s *t* test was used to compare means between two groups, and one-way or two-way ANOVA followed by Tukey’s or Bonferroni *post hoc* tests were used to determine significant differences among multiple groups. Differences were considered significant if *p* < 0.05. All data are expressed as mean ± SEM.

## Results

### Loss of Rgs7 in the striatum induces an antidepressant-like phenotype

**Figure 1.**
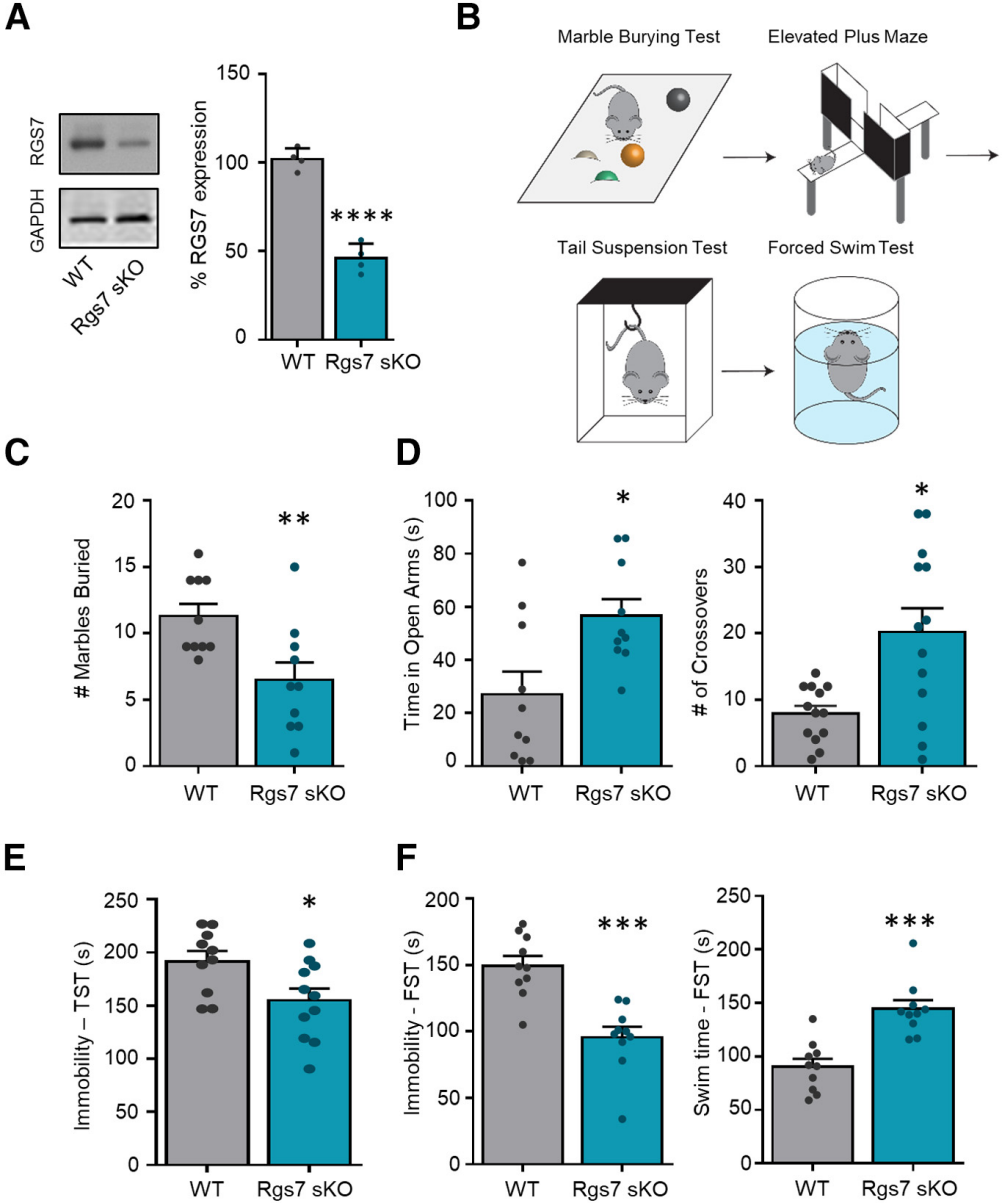
Ablation of striatal Rgs7 in mice results in an antidepressant-like phenotype. ***A***, Representative Western blottings and graphs of densitometry values for Rgs7 levels in the striatum of WT and Rgs7 sKO mice (*n* = 4/genotype). ***B***, Scheme of behavioral tests. WT and Rgs7 sKO mice were tested in (***C***) marble burying (MB), (***D***) elevated plus maze (EPM), (***E***) tail suspension test (TST), and (***F***) forced swim test (FST) (*n* = 10/genotype). Data shown as mean ± SEM; *p < 0.05, **p < 0.01, ***p < 0.001, ****p < 0.0001.

To study the role of striatal RGS7 in depression-related behaviors we eliminated Rgs7 in striatum by crossing conditional Rgs7^flx/flx^ strain with a striatal-specific driver Rgs9^cre^ mice to generate Rgs7^flx/flx^Rgs9^cre^ (Rgs7 sKO) and their wild-type littermates, Rgs7^flx/flx^ (WT; Fig. 1*A*). Mice were evaluated in a panel of behavioral tests to assess several aspects of anxiety-like and depressive-like behaviors including MB, EPM, TST, and FST (Fig. 1*B*). In the MB test, Rgs7 sKO mice displayed an anxiolytic-like phenotype as evident by burying fewer marbles (*t*_(18)_ = 2.999, *p* = 0.0077, *n* = 10/genotype; Fig. 1*C*). In the EPM, Rgs7 sKO mice spent more time in the open arm (*t*_(18)_ = 2.802, *p* = 0.018, *n* = 10/genotype) and increased number of crossovers into the open arm (*t*_(18)_ = 2.999, *p* < 0.05, *n* = 10/genotype; Fig. 1*D*). Rgs7 sKO mice also exhibited a reduced immobility time in the TST (*t*_(18)_ = 2.637, *p* = 0.017, *n* = 10/genotype; Fig. 1*E*). This antidepressant-like phenotype in the Rgs7 sKO mice was recapitulated in the forced swim test with a lower immobility time (FST, *t*_(18)_ = 4.993, *p* = 0 < 0.0001, *n* = 10/genotype) and a higher swim (mobility) time (*t*_(18)_ = 4.99, *p* = 0 < 0.0001, *n* = 10/genotype; Fig. 1*F*). In summary, the loss of striatal RGS7 induces an anxiolytic-like and antidepressant-like phenotype.

**Figure 2.**
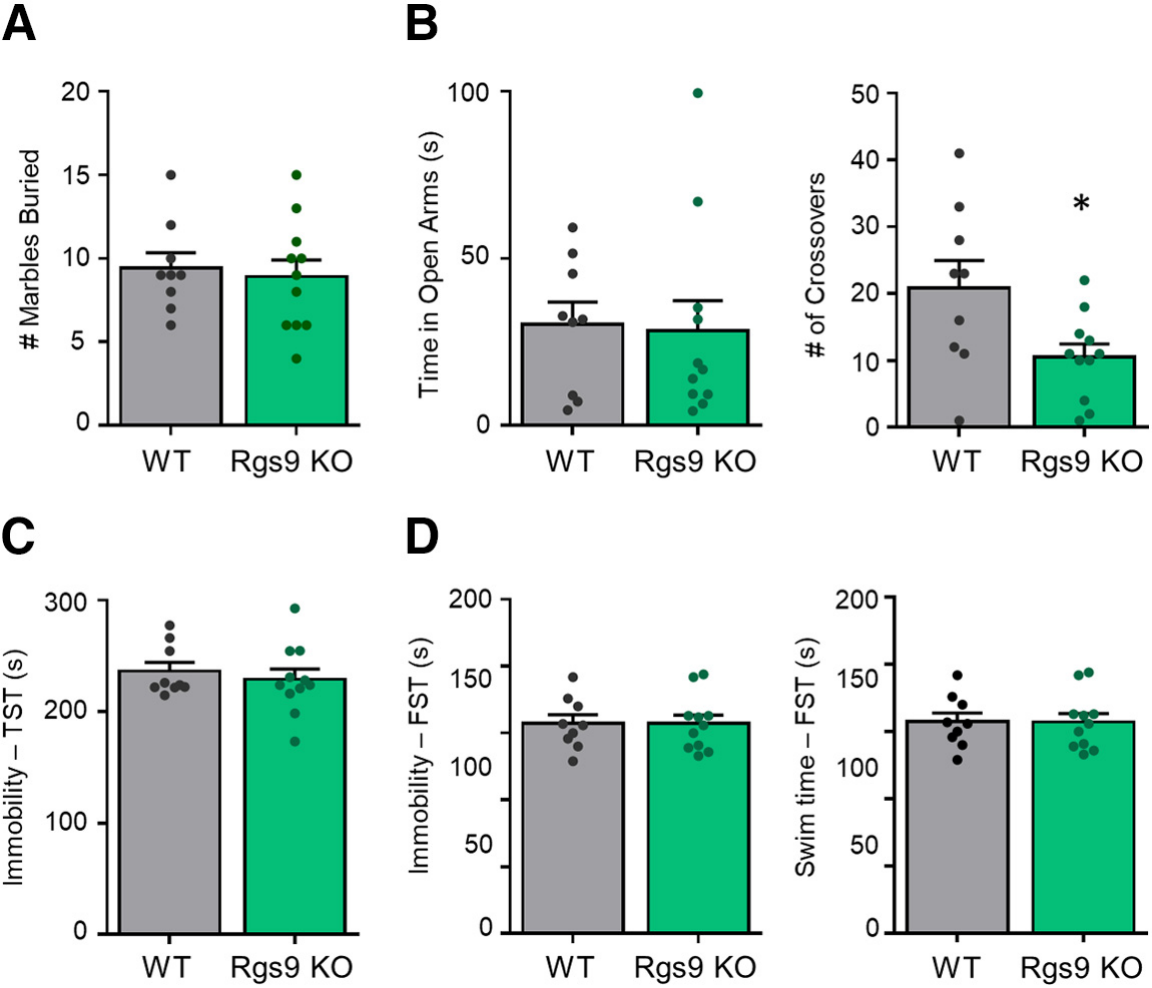
Elimination of Rgs9 does not influence behavior in acute stress procedures. Rgs9 knock-out mice were tested in (***A***) MB, (***B***) EPM, (***C***) TST, and (***D***) FST (*n* = 9–11 genotype). Data shown as mean ± SEM; *p < 0.05.

To address the behavioral selectively of Rgs7, we evaluated the role of Rgs9, a related member of the R7 RGS family, highly enriched in the striatum. In the MB test, there was no difference in the number of marbles buried between Rgs9 KO and their WT littermates (Fig. 2*A*). There was no difference in time spent in the open arm of the EPM but the number of crosses were decreased in the Rgs9 KO mice (*t*_(18)_ = 2.426, *p* < 0.05, WT *n* = 9 KO *n* = 11; Fig. 2*B*). Immobility times in the TST (Fig. 2*C*) and FST (Fig. 2*D*) were similar between Rgs9 KO and WT mice. Thus, loss of Rgs7, but not Rgs9, in the striatum selectively affects depression-related behaviors.

### Ablation of striatal Rgs7 does not influence behavioral responses to cocaine

**Figure 3.**
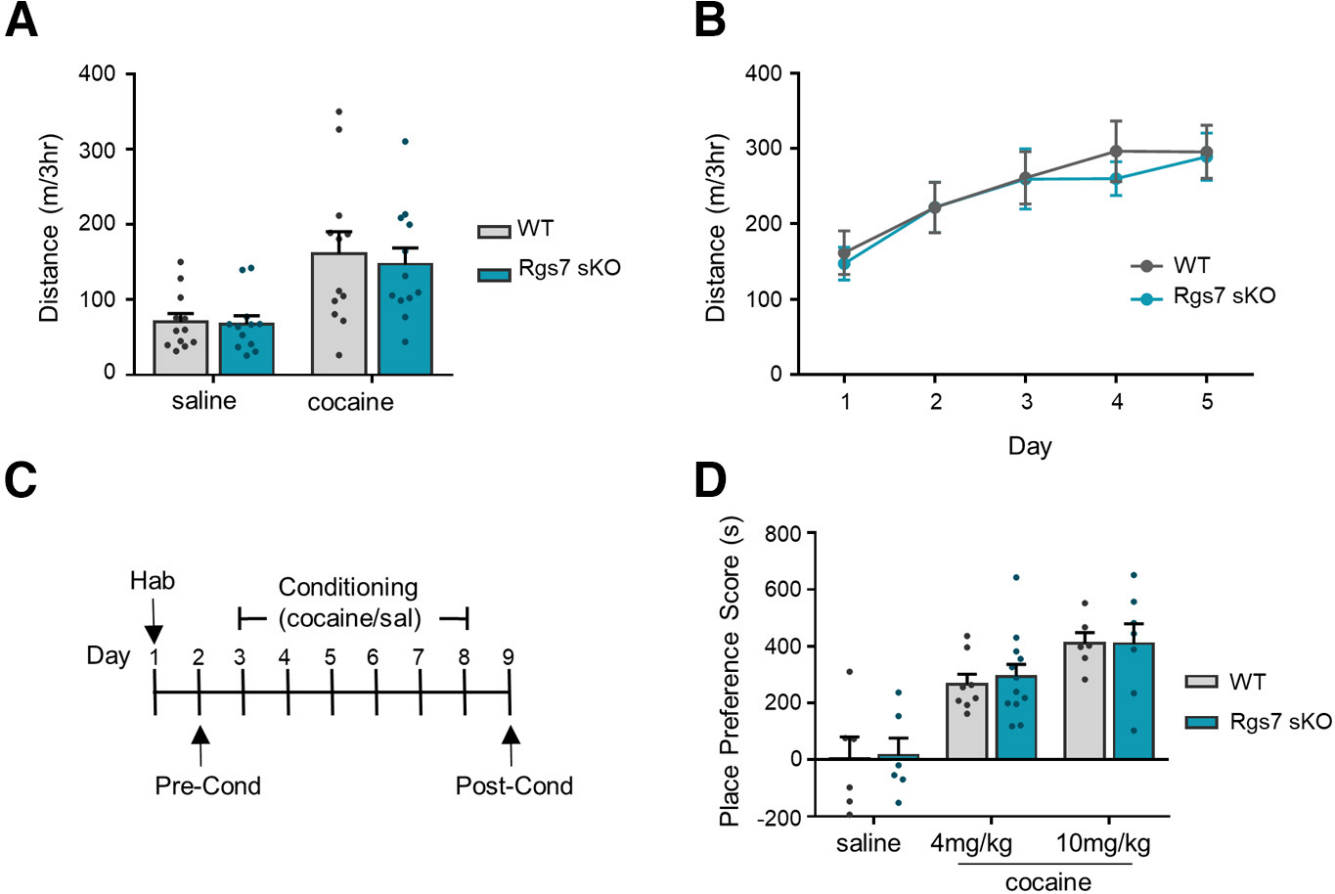
Elimination of striatal Rgs7 in mice does not affect cocaine-induced locomotion or CPP. ***A***, Total distance traveled for mice injected with saline or cocaine (15 mg/kg). ***B***, Total distance traveled for mice injected daily with cocaine for 5 d (*n* = 12 mice/genotype). ***C***, Timeline for CPP. ***D***, Effects of cocaine-induced CPP at doses of 4 and 10 mg/kg (*n* = 6–11/genotype). Place preference scores are calculated as the difference between time spent in the drug-paired side during postconditioning versus preconditioning tests. Data shown as mean ± SEM.

Previous studies implicated striatal Rgs7 in regulating the behavioral responses to morphine (Sutton et al., 2016). In order to determine whether this effect reflected general changes in reward setpoint common across drugs of abuse, we assessed the effects of cocaine administration in our Rgs7 sKO. In an open field arena, both WT and Rgs7 sKO mice showed increase in locomotor activity to cocaine as compared with saline (treatment *F*_(1,44)_ = 11.08, *p* = 0.018, *n* = 12/genotype; Fig. 3*A*). No significant difference between the genotypes was observed following cocaine administration. Locomotor activity was also examined following daily 5 d of cocaine administration and no difference between genotypes were found (Fig. 3*B*).

To test the rewarding effects of cocaine, CPP was conducted at doses of 4 and 10 mg/kg (Fig. 3*C*). As expected, cocaine administration induced a place preference at both doses that was observed by an increase in the time spent in the drug-paired compartment during the postconditioning phase compared with the preconditioning phase (treatment *F*_(2,39)_ = 23.85, *p* < 0.0001, *n* = 6–12/genotype; Fig. 3*D*). We found no significant difference in the place preference score between genotypes at either cocaine dose. Collectively, these results show that Rgs7 deficiency in striatal neurons does not alter cocaine-induced psychomotor activation, sensitization, or the rewarding properties of the drug.

### Elimination of striatal Rgs7 abolishes stress-induced reinstatement

**Figure 4.**
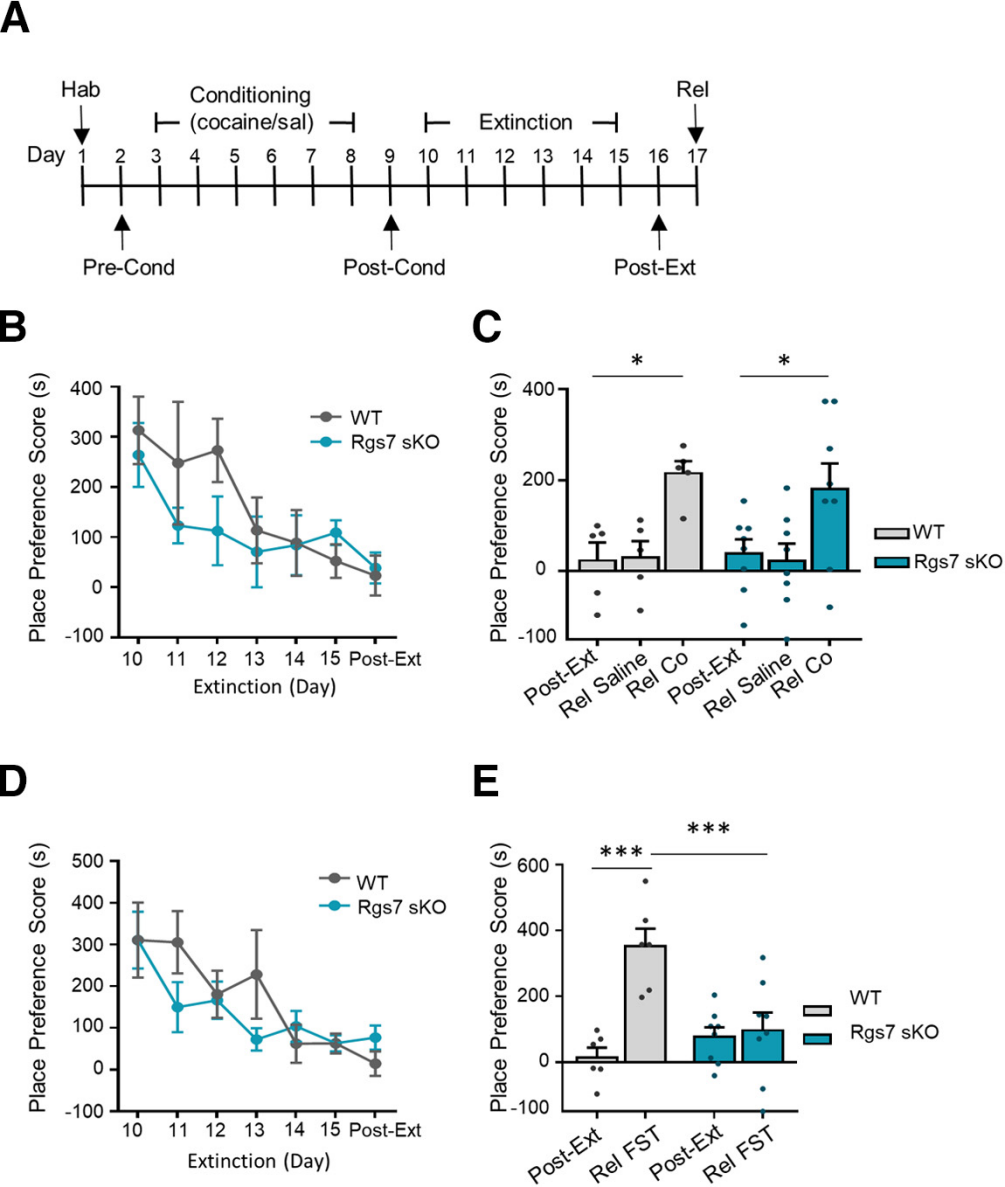
Ablation of striatal Rgs7 in mice display resiliency to stress-induced reinstatement. ***A***, Timeline for reinstatement. ***B***, Time course for extinction of cocaine CPP (*n* = 5–8 mice/genotype). ***C***, Cocaine reinstatement of extinguished cocaine-induced CPP (*n* = 5–8 mice/genotype). ***D***, Time course for extinction of cocaine CPP (*n* = 6–8 mice/genotype). ***E***, Force swim test reinstatement of extinguished cocaine-induced CPP. Data shown as mean ± SEM; *p < 0.05, ***p < 0.001.

Stress is a major factor influencing drug-seeking behaviors and as such we investigated the role of RGS7 in a stress-reinstatement of cocaine CPP (Fig. 4*A*). A 10 mg/kg cocaine dose was chosen to assess the role of RGS7 in stress-induced reinstatement. The place preference for cocaine was extinguished following 6 d of drug-free sessions where the time mice spent in the drug-paired compartment was similar between the postextinguished phase and the preconditioned phase (time *F*_(6,66)_ = 5.489, *p* = 0.0001, WT *n* = 5, KO *n* = 8; Fig. 4*B*). There was no difference between genotypes in the number of days to extinguish the place preference. To induce cocaine-reinstatement mice were subjected to a priming dose of cocaine or saline. Following the extinction of CPP, both WT and Rgs7 sKO mice were reinstated with cocaine and no difference between genotype was observed (treatment *F*_(2,22)_ = 10.78, *p* = 0.0005, WT *n* = 5, KO *n* = 8; Fig. 4*C*). There was no change in the place preference score with saline injection. A separate cohort of mice underwent extinction for cocaine CPP (time *F*_(6,72)_ = 7.246, *p* = 0.0001 WT *n* = 6 KO *n* = 8; Fig. 4*D*) and then were subjected to an acute stressor, a forced swim. The force swim stressor induced a place preference in WT but not in Rgs7 sKO mice (genotype *F*_(1,12)_ = 6.585, *p* < 0.01, treatment *F*_(1,12)_ = 12.06, *p* = 0.0046, interaction *F*_(1,12)_ = 9.745, *p* = 0.0088, WT *n* = 6, KO *n* = 8; Fig. 4*E*). Thus, loss of Rgs7 selectively protects mice from forced swim stress but not drug induced reinstatement of cocaine CPP.

### Effects of Rgs7 elimination on the proteome

**Figure 5.**
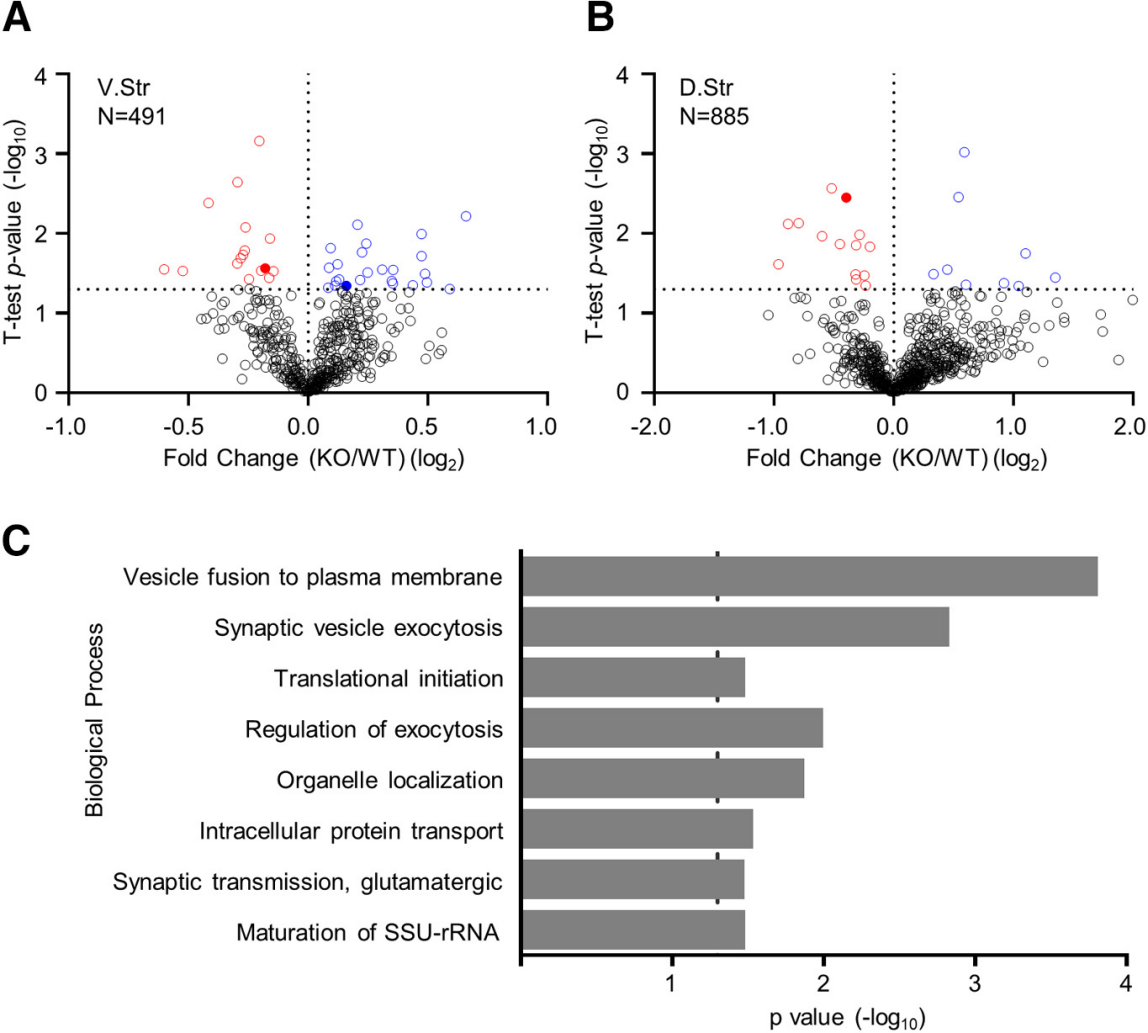
Proteomic analysis from conditional Rgs7 knock-out mice. Volcano plot showing the protein level fold change relative to significance between WT and Rgs7 sKO mice in the (***A***) dorsal and (***B***) ventral striatum. Significantly upregulated proteins are in blue (*p* < 0.05), significantly downregulated proteins are in red (*p* < 0.05), and all other proteins are in black (*n* = 5/genotype). Comparison of fold differences for all quantified proteins found in Extended Data Figure 5-1. ***C***, Panther analysis of statistically overrepresented biological processes in the ventral and dorsal striatum of Rgs7 sKO mice. Dotted line indicates Bonferroni corrected *p* = 0.05. Shown as a rank ordered list of most significant general biological processes.

To obtain insights into possible molecular underpinnings associated with the effect of striatal Rgs7 on behavior we identified proteins whose expression in the striatum was affected by the loss of Rgs7. This was achieved by carrying out a quantitative mass spectrometry of proteins in both the dorsal and ventral striatum. We found that 42 of 491 proteins in the ventral (Fig. 5*A*) and 23 of 885 proteins in the dorsal striatum (Fig. 5*B*) were significantly differentially expressed between WT and Rgs7 sKO mice (*p* values in the range 0.0499–6.9 × 10^−4^, Student’s *t* test, *n* = 5/genotype; Extended Data Fig. 5-1). To obtain insight into the processes affected by these changes, we explored association of proteins with significantly altered expression with functional networks using the Panther classification system. This analysis revealed that loss of striatal Rgs7 had a major effect on initiation of translation, vesicle fusion, and synaptic vesicle exocytosis (Fig. 5*C*). In particular, components of the eukaryotic initiation factor (eIF) complex, a cascade that regulates the initiation step in mRNA translation (Sonenberg and Hinnebusch, 2009) were differentially expressed in both regions of the striatum (Fig. 5*A*,*B*). Based on these results, we conclude that Rgs7 may exert many of its effects by controlling GPCR effects on protein biosynthesis and synaptic communication.

10.1523/ENEURO.0365-20.2020.f5-1 Extended Data Figure 5-1 Fold differences between WT and Rgs7 sKO mice for the proteomic analysis. Table listing all quantified proteins between WT and Rgs7 sKO mice in the dorsal and ventral striatum. Columns are arranged left-to-right as protein accession number, gene name, fold change (KO vs WT), Log_2_ of fold change, Student’s *t* test, -Log_10_ of *t* test, and protein description. Log_2_ fold change and -Log_10_ *t* test are the values graphed in the volcano plots of Figure 5*A*,*B*. Download Figure 5-1, XLSX file.

## Discussion

The current study demonstrates the contribution of striatal Rgs7 toward depression-related behaviors and their relevance to substance abuse. Our behavioral experiments show that the lack of RGS7 in the striatum results in an antidepressant-like and anxiolytic-like phenotype but does not affect cocaine-induced locomotion, sensitization or CPP. Furthermore, striatal specific ablation of Rgs7 resulted in a resiliency to stress reinstatement of previously extinguished cocaine CPP but not following re-exposure to a priming dose of the cocaine. We also found that elimination of Rgs9, a highly related and abundant RGS protein in the same neuronal populations produced no behavioral effects in the depressive-like assays. These observations suggest that the reactions that lead to the development of the phenotype are specifically controlled by the Rgs7. Overall, the results reveal a prominent contribution of striatal neurons controlled by Rgs7 to depressive-like behaviors and stress-induced reinstatement.

We have previously found that the elimination of Rgs7 in the PFC was sufficient to drive antidepressant-like and anxiolytic-like phenotype using the same behavioral tests (Orlandi et al., 2019). Current results complement these findings and demonstrate the ability of Rgs7 to act across different brain circuits to regulate affective behaviors. Perhaps it is not entirely surprising that our results revealed no regional specificity of Rgs7 effects as both the PFC and striatum are interconnected and involved in mediating mood and emotionality. While the exact molecular mechanism underlying the observed behavioral effects remains to be determined, it is known that Rgs7 acts as a negative regulation of Gαi/o-coupled GPCRs (Anderson et al., 2009b). Studies with a global knock-out of Rgs7, implicated both of α2A-adrenergic and GABAB receptors as mediators of antidepressant phenotypes (Orlandi et al., 2019). This suggests that multiple GPCRs may play a role in this process, and it would be of interest to explore which GPCR system drives the striatal phenotype.

Given that RGS proteins are direct regulators of GPCR signaling, there has been a forthcoming effort to study their role in the etiology and treatment of depression (Senese et al., 2018), and our study adds to this knowledge. Our genetic manipulations allow for a direct comparison of Rgs7 and Rgs9 in the same neuronal population allowing us to conclude that they have distinct behavioral profiles within the striatum and do not compensate for each other. The other brain-enriched member of the R7 family, Rgs6, has also been implicated in mood regulation. Global Rgs6 knock-out mice display antidepressant-like behaviors and this phenotype was reversed by serotonin 5-HT1A receptor antagonist pretreatment (Stewart et al., 2014). However, treatment with 5-HT1A antagonist has been shown to be ineffective toward the antidepressant-like phenotype in a model of Rgs7 (Orlandi et al., 2019). Although these members of the R7 family all target Gαi/o (Posner et al., 1999; Hooks et al., 2003), share common binding partners (Cabrera et al., 1998; Makino et al., 1999; Zhang and Simonds, 2000; Martemyanov et al., 2005), and are expressed in the striatum (Thomas et al., 1998; Rahman et al., 1999; Anderson et al., 2009a), there appears to be a selectivity for the Gαi/o-coupled GPCR and consequently produces different phenotypic outcomes (Anderson et al., 2009b). This nonredundant function of RGS-mediated behaviors has been observed in other behavioral paradigms and it is intriguing how selective Rgs7 is toward Gi/o-coupled GPCR signaling (Zachariou et al., 2003; Anderson et al., 2010; Sutton et al., 2016). Furthermore, it appears that the loss of one R7 member is not compensated by other members of the family, although they are all expressed in the same striatal neurons. In agreement with this, the elimination of Rgs7 does not affect the levels of Rgs6 or Rgs9 and thus we attribute our observed behavioral effects to the loss of Rgs7 expression (Sutton et al., 2016).

In this study, the driver line Rgs9^cre^ was used to target striatal neurons, as expression of Cre recombinase has been shown to be restricted to postsynaptic neurons in the striatum (Sutton et al., 2016; Tecuapetla et al., 2016). Western blottings showed a substantial decrease of striatal Rgs7 protein with residual amounts likely from glial cells that do not express Cre and/or from incoming projection from the VTA, cortex, and other brain regions (Dang et al., 2006). Furthermore, this knock-out strategy does not discriminate between MSNs and cholinergic interneurons. As Rgs7 is expressed in these neuronal populations, we cannot fully address the cell-specific contributions of Rgs7 toward depression-like behaviors. While future studies are needed to parse out the cell-specific roles of Rgs7 in the striatum, it appears striatal Rgs7 is a molecular determinant to drive stress-related behaviors.

The behavioral paradigm to assess depressive-like behaviors allowed us to evaluate an individual animal across complementary tests (MB, EPM, TST, and FST). This multimodal approach has been shown to reduce behavioral variability across several tests and allow for a robust and comprehensive characterization for an individual mouse (Crawley and Paylor, 1997; Guilloux et al., 2011). Although the order across multiple days of testing is designed to mitigated stress (least to the more stressful test), we cannot rule out that conducting several tests could influence behavioral outcomes.

Stress and drug re-exposure are common precipitating factors for relapse in recovering cocaine addicts. Although both stimuli can trigger drug relapse, they do not necessarily require activation of overlapping neurobiological pathways (Kalivas and McFarland, 2003). Significant effort has been made to dissect the mechanism involved in stress and drug cued relapse. For example, metabotropic glutamate receptors have been implicated in cocaine priming and reinstatement (Baker et al., 2003; Kupchik et al., 2012), where both mGluR2/3 and mGluR5 inhibition in the NAc have been shown to prevent cocaine reinstatement (Kumaresan et al., 2009; Mahler et al., 2014). Targeting CREB signaling in the NAc affected stress reinstatement but failed to augment drug induced reinstatement (Kreibich and Blendy, 2004; Briand et al., 2010). CREB is activated by the cAMP pathway and we have found that Rgs7 KO mice have an increase in cAMP levels (Orlandi et al., 2019). Our findings that Rgs7 is a mediator of stress reinstatement but not for cocaine agree with previous studies that have demonstrated dissociable mechanisms of pharmacological and stress reinstatement (Mantsch et al., 2010; Nair et al., 2013). In addition to Rgs7 being a mediator of stress reinstatement, it also prevents stress-induced depression (Orlandi et al., 2019). This raises an intriguing notion that Rgs7 may be a general regulator for stress-related behaviors.

Our results also provide interesting insights into changes in striatal proteome induced by the loss of Rgs7. Notably, our proteomic screen revealed several eIFs that were significantly differential expressed in striatal tissues lacking Rgs7. The eIF complex is considered to be the rate limiting step in protein synthesis tightly regulating this fundamental cellular process (Sonenberg and Hinnebusch, 2009). A growing body of evidence has implicated the importance of eIF in normal neuronal cell function (Amorim et al., 2018). Inhibition of this process induces depressive-like behaviors in rodents, and downregulation of several eIF proteins have been detected in MDD patients (Jernigan et al., 2011; Yang et al., 2013; Aguilar-Valles et al., 2018). Furthermore, ketamine and traditional antidepressants affect local protein synthesis and this action is sufficient to ameliorate depressive-like behaviors (Park et al., 2014b; Liu et al., 2015). While further investigation of Rgs7 signaling is warranted, it is plausible that Rgs7 influence on protein synthesis drives depressive-like responses.

In summary, our data demonstrates that Rgs7 plays a prominent role in depression and the regulation of stress-induced reinstatement of cocaine CPP. Together these finding may provide a better understanding for the molecular mechanism involved in resiliency to the maladaptive effects of stress.

## Synthesis

Reviewing Editor: Christie Fowler, University of California, Irvine Decisions are customarily a result of the Reviewing Editor and the peer reviewers coming together and discussing their recommendations until a consensus is reached. When revisions are invited, a fact-based synthesis statement explaining their decision and outlining what is needed to prepare a revision will be listed below. The following reviewer(s) agreed to reveal their identity: Sunila Nair, Megan Fox.

Both the reviewers and the editor felt that the manuscript was of high interest for the field. This manuscript characterizes “affective-like” and drug-related behaviors in mice lacking Rgs7 in striatal neurons. It was further noted that the proteomics data will be a valuable resource and should launch additional investigations into the causal role of different striatal proteins in stress and drug-related behaviors. In the consultation session, it was concluded that additional studies are necessary to fully support the manuscript’s main conclusions. First, additional control experiments should include (1) comparing the stress condition to no stress condition and (2) comparing cocaine to a saline control condition. A second point of major concern was the lack of validation of the knockout strategy in the mice. Finally, it was unclear as to whether the knockout mice fully extinguish their behavior, and if not, the conclusions of the study may not be fully supported.

Reviewer 1:

This is an interesting study examining the role of Rgs7 in the striatum using a genetic mouse model. Using a battery of behavioral tests, the authors found that a lack of Rgs7, but not Rgs 9 in the striatum produces an anxiolyic and antidepressant phenotype. The authors also found that the lack of striatal Rgs 7 attenuates stress-induced, but not cocaine-priming induced reinstatement of cocaine CPP while having no effect on cocaine CPP and cocaine-induced locomotor activity.

Major issue

The main finding of this manuscript is the role of Rgs7 in cocaine reinstatement. My major criticism is with the design of this experiment. All animals are exposed to the stressor (or cocaine, in case of cocaine-priming reinstatement) without any control condition (No stress, or saline for cocaine-prime). The lack of these data makes the conclusions drawn from this study problematic.

Minor concerns-

- Title: The title does not convey to the readers the authors’ findings on the role of Rgs 7 on anxiety and depressive behaviors.

- In the ‘Animals’ section please specify the total ‘n’ of animals used in all studies. Please specify the ‘n’ for each experiment in the Results section.

- Please provide data for swimming time in addition to immobility time in the forced swim test. Was the pre-test and test performed on consecutive days?

- Please remove ‘morphine’ from the Methods (CPP experiment), since this is a cocaine study.

- Since it appears that the behavioral tests were performed serially in the same mice it is possible that exposure to a specific stimulus influenced the behavioral outcomes of the subsequent experiments. Please acknowledge this in the discussion section.

Reviewer 2:

In this manuscript, the authors characterize “affective-like” and drug-related behaviors in mice lacking Rgs7 in Rgs9 expressing (striatal) neurons. They find a behavioral phenotype consistent with global Rgs7 knockout-i.e. “stress-resilient” and decreased “anxiety-like” behavior. Cocaine-induced hyperlocomotion and conditioned place-preference are intact in the KO’s, but the Rgs7 KO mice are resistant to swim-stress induced reinstatement of CPP. The authors then assess changes to the striatal proteome in the KO mice under baseline conditions. Overall, the manuscript is well-written and of interest to both the stress and addiction fields, and the proteomics will be a valuable resource to launch additional investigations. However, I have some concerns with the manuscript, including language I feel is “too strong.” The title in particular, should be revised to specify reinstatement of cocaine conditioned place preference. My specific comments are below.

1. The overall strategy for striatal knockout of Rgs7 is solid. Rgs9 is indeed, enriched in the striatum, but there is also non-trivial Rgs9 expression in the PAG and spinal cord. It is important to demonstrate (via ISH/IHC, or WB/qPCR) the extent to which Rgs7 is depleted in the striatum, and intact in the rest of the brain.

2. This study is conducted solely in male mice. I appreciate that not all studies can include sex as a biological variable, especially in COVID times. However, it should be emphasized throughout the text, especially in the abstract, that these findings are from exclusively male mice.

3. “We report that inactivation of Rgs7 specifically in striatal neurons results in prominent antidepressant effects and protects mice from stress-induced but not drug-cued reinstatement of cocaine craving.” This language is too strong. CPP does not measure cocaine craving, and FST/EPM does not measure depression.

4. The figure 2 legend indicates Rgs6 knockout, while the text and figure indicates Rgs9. Fig2 legend also states 8-9 mice per genotype, but this is incompatible with the reported t statistic t(18)

5. “Stress-induced reinstatement” typically refers to reinstatement of drug-seeking in the context of drug self-administration, often elicited by footshock. Since this manuscript uses CPP, the phrase should be revised to “reinstatement of cocaine CPP.” Also, the Rgs7 sKO mice show this anti-stress behavioral phenotype. How are you certain that the swim stress is actually stressful to these mice? In the absence of physiological measurements of stress (e.g. cort) it is difficult to call this a true “Stress-induced reinstatement.” I suggest “swim-stress fails to induce reinstatement of cocaine CPP in rgs7 sKO mice"

6. The description for when mice were considered to have extinguished place preference is confusing. “Preference for the drug side was considered extinguished when the change in time spent in the cocaine side relative to preconditioning values minus the standard error of the mean was below zero”. -the SEM of what??

7. The Rgs7 sKO mice don’t seem to have extinguished as well as the wildtype mice in Fig 4C. It is difficult to tell if genotype has any bearing on CPP extinction, since a range of n’s is reported, and there are no individual data points on the graphs. Please report how many mice per genotype extinguished vs did not extinguish.

8. Please report which proteins belong to the overrepresented GO terms shown in 5C. Are these GO terms overrepresented in both dorsal and ventral striatum? Do they differ if analyzed separately?

9. Page 6- in describing the cocaine CPP, there is mention of morphine.

10. Can the authors speculate which striatal neurons contribute to the behavioral phenotype of the Rgs7 sKO mice? Does it exert effects in medium spiny neurons, interneurons, or both?
